# Stability Subtypes of Callous–Unemotional Traits and Conduct Disorder Symptoms and Their Correlates

**DOI:** 10.1007/s10964-016-0520-4

**Published:** 2016-06-14

**Authors:** Hedwig Eisenbarth, Chara A. Demetriou, Melina Nicole Kyranides, Kostas A. Fanti

**Affiliations:** 1 Department of Psychology, University of Southampton, University Road, Southampton, SO17 1BJ UK; 2 Department of Psychology, University of Cyprus, Kallipoleos 65, 1 Panepistimiou Avenue, 2109 Aglantzia, Nicosia Cyprus

**Keywords:** Conduct disorder, Callous–unemotional traits, Aggression, Stability, Anxiety

## Abstract

Callous–unemotional traits and conduct disorder symptoms tend to co-occur across development, with existing evidence pointing to individual differences in the co-development of these problems. The current study identified groups of at risk adolescents showing stable (i.e., high on both conduct disorder and callous–unemotional symptoms, high only on either callous–unemotional or conduct disorder symptoms) or increasing conduct disorder and callous–unemotional symptoms. Data were collected from a sample of 2038 community adolescents between 15 and 18 years (1070 females, *M*_age_ = 16) of age. A longitudinal design was followed in that adolescent reports were collected at two time points, 1 year apart. Increases in conduct disorder symptoms and callous–unemotional traits were accompanied by increases in anxiety, depressive symptoms, narcissism, proactive and reactive aggression and decreases in self-esteem. Furthermore, adolescents with high and stable conduct disorder symptoms and callous–unemotional traits were consistently at high risk for individual, behavioral and contextual problems. In contrast, youth high on callous–unemotional traits without conduct disorder symptoms remained at low-risk for anxiety, depressive symptoms, narcissism, and aggression, pointing to a potential protective function of pure callous–unemotional traits against the development of psychopathological problems.

## Introduction

Investigating the co-occurrence between callous–unemotional traits (i.e., low guilt, low empathy) and conduct disorder symptoms (i.e., vandalism, bullying, stealing) can aid in the identification of more homogeneous groups of individuals, providing important insights informing the etiology of antisocial behavior (Fanti 2013). The presence or absence of callous–unemotional traits designates unique subgroups of children with conduct disorder, scoring on opposite extremes on measures of anxiety and fear (Fanti et al. 2016b). Further, developmental stability of callous–unemotional traits has been associated with severe and persistent aggressive and antisocial behavior compared to groups showing developmental instability in these traits (Fanti and Centifanti 2014). In addition, increases or decreases in callous–unemotional traits during childhood have been linked to similar changes in contextual, behavioral and individual problems, providing evidence for potential risk and protective factors (Fanti et al. 2016b). The current study aims to investigate the co-development of conduct disorder symptoms and callous–unemotional traits during adolescence, and compare the identified subgroups on measures of internalizing problems, aggressive behavior, personality traits, peer relationships, and media violence exposure. Identifying differences between groups of adolescents showing stability or instability in conduct disorder and callous–unemotional traits can inform intervention efforts during a developmental stage marked with increases in antisocial behavior (Moffitt 1993).

### Subtypes Based on Symptom Stability

Studies investigating the co-development of conduct disorder symptoms and callous–unemotional traits during childhood identified distinct stability subtypes. A study that followed children from age 7–12 provided evidence that children with stable conduct disorder symptoms were divided into those with stable, increasing, and decreasing callous–unemotional traits (Fontaine et al. 2011). Similar groups have been identified in a different sample of community children of approximately the same age span, with stable, increasing, and decreasing callous–unemotional subtypes showing the same developmental trajectories in conduct disorder symptomatology (Fanti et al. 2016a). Adding to this evidence, a study conducted with preschoolers pointed to the existence of two stable subgroups scoring high on conduct disorder symptoms with low or high callous–unemotional traits and two unstable groups showing decreases or increases in both conduct disorder symptoms and callous–unemotional traits (Klingzell et al. 2015). Among children with conduct disorder symptoms, those high on callous–unemotional traits showed higher severity in antisocial behavior (Klingzell et al. 2015).

Identifying similar stable or unstable conduct disorder and callous–unemotional subtypes during adolescence can inform the co-development of these symptoms across different developmental stages. However, only limited information is available in terms of the existence of these stability subtypes during adolescence. Kyranides, Fanti and Panayiotou (2016) identified a group of adolescents with only elevated stable callous–unemotional traits (8.9 %), a group with stable high conduct disorder symptoms and callous–unemotional traits (5.7 %), a group with only elevated stable conduct disorder symptoms (4.9 %), as well as a group that was characterized by increases in callous–unemotional and conduct disorder symptoms from time 1 to time 2 (5.4 %). Compared to studies conducted with children, Kyranides et al. (2016) did not identify a decreasing conduct disorder and callous–unemotional symptoms group, providing evidence for greater stability during the adolescent developmental period. The identification of an increasing group agrees with evidence that conduct disorder symptoms increase in mid adolescence (Kyranides et al. 2016). However, in this specific study, the identified groups have not been compared on individual, contextual, and behavioral variables that might inform treatment efforts, which is an aim of the current study.

### Differentiating Subtypes by Internalizing Factors

Theoretically, low anxiety and depression is a characteristic of youth high on callous–unemotional traits, whereas high anxiety and depression characterizes children and adolescents scoring high on antisocial behavior and low on callous–unemotional traits (Frick and Ellis 1999). Indeed, children high on conduct problems alone were found be characterized by anxiety and increased physiological reactions to emotional stimuli compared to those high on callous–unemotional traits and normal controls (Fanti et al. 2016b). Contrary to these findings, prior work also suggested that children and adolescents high on both conduct problems and callous–unemotional traits were not differentiated from children high on conduct problems alone or with increasing callous–unemotional traits based on questionnaire measures of anxiety and emotional problems (Fanti 2013; Fontaine et al. 2011). There is even more inconsistency in findings on the association between anxiety/depression and callous–unemotional traits reporting that this association is either nonexistent (Fanti et al. 2013), negative (see for a review: Feilhauer and Cima 2013) or positive (Frick et al. 2014). Taking stability subtypes into account might provide more detailed evidence to understand these inconsistent findings.

### Differentiating Subtypes by Aggression

Callous–unemotional traits and conduct problems have been found to be related to distinct forms of antisocial behavior, including proactive and reactive aggression (Fanti et al. 2013). Proactive aggression is defined as a planned, controlled, and purposeful execution of an aggressive act with the aim of achieving a desired goal. In contrast, reactive aggression is triggered in response to real or perceived provocation and is emotionally charged and under-controlled (Dodge et al. 1990; Mathias and Stanford 2003; Raine 2002; Scarpa et al. 2008). In addition, proactive aggression has been linked to affective and callous–unemotional psychopathic traits, low anxiety and distress, while reactive aggression to impulsive-antisocial psychopathic traits, high anxiety and distress (Blair 2001; Helfritz and Stanford 2006; Patrick and Zempolich 1998; Raine et al. 2006; Stanford et al. 2008; Scarpa et al. 2010). Taking these findings into account, proactive aggression might be more likely to be expressed by antisocial youth scoring high on callous–unemotional traits, while reactive aggression by antisocial youth scoring low on these traits. However, both proactive and reactive forms of aggression were found to be elevated among youth high on callous–unemotional traits in studies conducted with juvenile offenders and community samples of adolescents (Longman et al. 2015). Further, individuals showing a combination of callous–unemotional traits and conduct problems score higher on both proactive and reactive aggression compared to individuals scoring high on conduct problems alone (Frick et al. 2003). Unfortunately, no prior work compared stability subtypes in terms of these forms of aggression, which is an aim of the current study.

### Differentiating Subtypes by Personality Variables

Callous–unemotional traits constitute one dimension of personality traits being captured under the psychopathy construct. A psychopathic personality further includes interpersonal (e.g., narcissism) and behavior-related (e.g., sensation seeking, impulsivity) personality traits (e.g. Andershed et al. 2002; Frick and Hare 2001). Both interpersonal and behavioral psychopathic traits were found to be higher in antisocial children who exhibit stable high or increasing callous–unemotional traits compared to antisocial children with decreasing or low callous–unemotional traits (Fanti 2013; Fontaine et al. 2011; Klingzell et al. 2015). In fact, in children, stability and change in callous–unemotional traits followed the same pattern of stability and change in narcissistic and impulsive traits (Fanti et al. 2016a). Importantly, despite their high scores on narcissism, individuals high on callous–unemotional traits tend to have low self-esteem (Fanti 2013), and this combination has been described as a vulnerable self-esteem (Brummelman et al. 2016). In fact, the combination of high narcissism and low self-esteem has been associated with engagement in severe antisocial behavior (Fanti and Henrich 2015), which characterizes youth high on both conduct disorder symptoms and callous–unemotional traits. These findings are expected to be replicated during adolescence.

### Differentiating Subtypes by Environmental Factors

Contextual variables and their association with stability subtypes of conduct disorder symptoms and callous–unemotional traits have been mainly investigated in terms of parenting variables. The influence of self-perception within the peer group, which is relevant to the adolescent developmental period, has not been investigated thoroughly. These associations are of great importance because problematic peer relationships have been found to predict conduct disorder behaviors among adolescents (Kahn et al. 2013). Limited evidence suggest that callous–unemotional traits are related to deviant peer group selection (Kyranides et al. 2016) and low support from peers (Fanti 2013). In addition, high callous–unemotional traits, irrespective of anxiety and conduct disorder symptoms, were associated with low peer conformity, high popularity striving, and high peer pressure (Fanti et al. 2013). These findings indicate that adolescents high on callous–unemotional traits with or without co-occurring conduct disorder symptoms might be equally likely to report problematic peer relationships.

Violent media exposure might be an additional contextual variable associated with the development of callous–unemotional traits and conduct disorder symptoms. Indeed, studies with adolescent offenders or adolescents within the community found that exposure to violence explained the association between callous–unemotional traits and antisocial behavior (see for a review: Feilhauer and Cima 2013). Further, adolescents with co-occurring conduct problems and callous–unemotional traits were more likely to be exposed to media violence compared to adolescents high on conduct problems or callous–unemotional traits alone (e.g. Fite et al. 2009; Fanti et al. 2013). However, there is yet no evidence for differences between stability subtypes.

## Current Study and Hypotheses

Prior work provides evidence for heterogeneous groups of children and adolescents differentiated on their levels of conduct disorder symptoms and callous–unemotional traits (e.g., Fanti 2013). These groups have been distinguished on individual problems and contextual maladjustment. However, it is unclear how stability and change in both callous–unemotional traits and conduct disorder symptoms during adolescence relate to aggressive behavior, internalizing (anxiety, depression) problems, personality traits, such as narcissism, impulsivity, self-esteem, and sensation seeking, and contextual variables, including peer perception and violent media exposure.

Adding to prior longitudinal work, this study investigates the characteristics of different callous–unemotional traits and conduct disorder symptom stability subtypes identified across a period of 1 year in a large community sample of adolescents. Four at risk groups will be compared: a group with stable high conduct disorder symptoms and callous–unemotional traits, a group high only on callous–unemotional traits, a group high only on conduct disorder symptoms, and a group with increasing conduct disorder symptoms and callous–unemotional traits. We hypothesize that youth with stable high conduct disorder symptoms irrespective of callous–unemotional traits will score high on proactive and reactive aggression across time, while youth high on callous–unemotional traits, irrespective of conduct disorder symptoms, will report problems with peers and low self-esteem. We further expect that youth with stable conduct disorder symptoms and callous–unemotional traits will score high on measures of internalizing problems, narcissism and impulsivity. Youth in the group with high callous–unemotional traits alone are expected to be at low risk with regard to aggressive behavior and internalizing symptoms. Finally, we expect the group with increasing conduct disorder symptoms and callous–unemotional traits (increasing group) to be a unique group of youth demonstrating increases in individual and contextual maladjustment across adolescence.

## Methods

### Sample

The sample consists of *N* = 2067 adolescents living in the Republic of Cyprus. After excluding those with incomplete data, data from 2023 adolescents were included in the analysis (1070 female, 953 male). Adolescents ranged in age between 15 and 18 years at the initial assessment (*M*_age_ = 16, *SD* = .89) and data were collected from high school students in grades 10 (39 % of the sample), 11 (31.5 %) and 12 (29.5 %). The sample was diverse in terms of parental education levels: 17.61 % did not complete high school, 47.89 % had a high school education and 34.5 % had a higher education degree, which is representative of the population in Cyprus. These data have been analyzed to identify subsamples of individuals at high risk for callous–unemotional traits and part of the sample has been included in an experimental study (Kyranides et al. 2016).

Following approval of the study by the Centre of Educational Research and Assessment (CERE), Pedagogical Institute, Ministry of Education and Culture, twelve high schools in three provinces (Nicosia, Limassol and Larnaca) were randomly selected for participation. Parents were informed of the longitudinal nature of the study and 96 % of those contacted consented to their child’s participation. In both assessments, students completed a battery of questionnaires used in the latent profile analysis and Analysis of Variance (ANOVA). A high percentage of students in the original sample (98.6 %) participated in the follow-up assessment 1 year later. Attrition was due to an inability to contact students who had relocated or transferred to a different school.

**Fig. 1:**
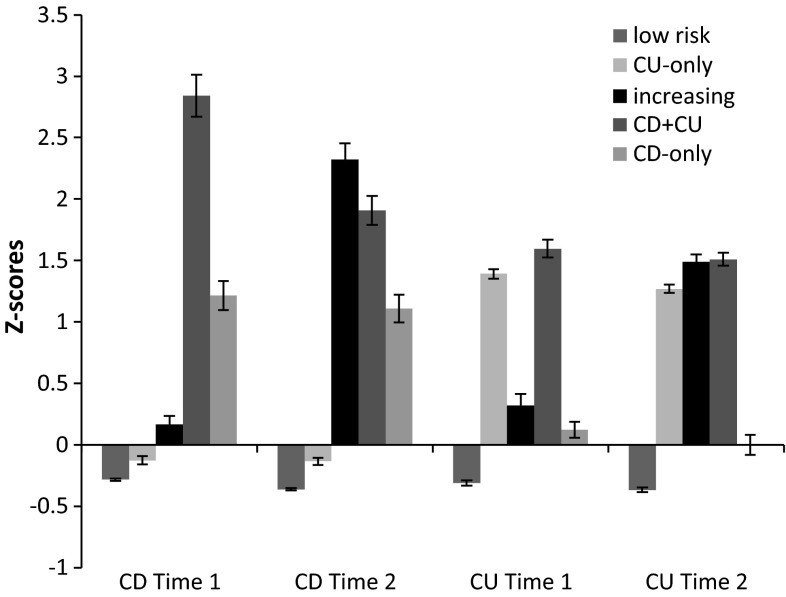
Callous–unemotional traits (CU) and conduct disorder symptoms (CD) scores (z-scored) at Time 1 and Time 2 for the 5 groups: “low” (low on conduct disorder symptoms and callous–unemotional traits), “CU-only” (high on callous–unemotional traits, low on conduct disorder symptoms), “increasing” (low on both at Time 1, high on both at Time 2), “CD + CU” (high on both conduct disorder symptoms and callous–unemotional traits) and “CD-only” (high on conduct disorder symptoms, low on callous–unemotional traits): means and standard errors of the mean

As described in Kyranides et al. (2016), using Latent Profiles Analysis five distinct groups were identified, which are depicted in Fig. 1. The majority of the sample scored below average on the two measures under investigation (“low” group, 61.4 % females). Youth in the group with callous–unemotional traits alone (36.7 % females) scored high on callous–unemotional traits across time, but below average on conduct disorder symptoms. Adolescents in the group with increasing traits (15.3 % females) exhibited increases in levels of callous–unemotional traits and conduct disorder symptoms from time 1 to time 2. Adolescents in the group with combined conduct problem symptoms and callous–unemotional traits (13.2 % females) were differentiated from the rest of the groups by their continuous high scores on both conduct disorder symptoms and callous–unemotional traits. Finally, youth in the group with conduct disorder symptoms alone (30.2 % females) scored high on conduct disorder symptoms across time, but at average levels on callous–unemotional traits. These groups will be used in the analysis.

### Measures

#### Callous–Unemotional Traits (Time 1 and 2)

The Inventory of Callous–Unemotional Traits (ICU; Frick 2004) is designed to assess self-reported callous–unemotional traits in youth. The ICU comprises of 24 items (e.g., “What I think is “right” and “wrong” is different from what other people think”) that are rated on a 4-point Likert-scale ranging from 0 (not at all true) to 3 (definitely true). Item scores are summed to form a total score that demonstrated adequate internal consistency in the present study, *α* = .77–.80. Previous research has provided evidence for the validity of ICU scores in community and high risk samples of adolescents (Essau et al. 2006; Fanti et al. 2009; Kimonis et al. 2008).

#### Conduct Problems, Anxiety (Time 1 and 2) and Depressive Symptoms (Time 2)

The Checkmate plus *Youth’s Inventory*-*4* (YI-4; Gadow and Sprafkin 1999) is a self-report checklist of DSM-IV symptomatology for the most common disorders of childhood and adolescence. Youth rate YI-4 symptoms on a 4-point Likert scale ranging from 0 (never) to 3 (very often). For the present study only the 15-items corresponding to Conduct Disorder symptoms (e.g., “I stay out at night when I am not supposed to”; α = .88–.90), the 6-items corresponding to Anxiety symptoms (e.g., “I have trouble getting myself to stop worrying”; α = .84–.85), and 11-items corresponding to Depressive symptoms (e.g., “I feel unhappy or sad”; α = .77) were used in the analyses. The items were summed to create overall conduct disorder, anxiety and depression subscales. Previous research has provided evidence for convergent and discriminant validity of the YI-4 in community and clinical samples of adolescents (Gadow et al. 2002, 2004; Fanti et al. 2013).

#### Self-Esteem (Time 1 and 2)

The *Rosenberg Self*-*Esteem Scale* (RSES; Rosenberg 1965) is a 10-item measure of global self-esteem. Individuals report on their current feelings on a five-point Likert scale ranging from 0 (strongly disagree) to 3 (strongly agree). Five items are worded positively (e.g., “On the whole, I am satisfied with myself”) and five are worded negatively (e.g., “At times, I think I am no good at all”). RSES items are summed to form a total score with higher scores indicating higher self-esteem (α = .70–.73).

#### Impulsivity (Time 1) and Narcissism (Time 1 and 2)

The *Antisocial Process Screening Device*-*Youth Version* (APSD; Frick and Hare 2001) is a self-report rating scale designed to assess dimensions of psychopathy among youth. APSD items are rated on a three-point Likert scale ranging from 0 (not at all true) to 2 (definitely true). For the present study, only the 5 items corresponding to the Impulsivity (e.g., “I do not plan ahead or leave things until the last moment”; α = .65–.69) and the 7 items corresponding to the Narcissism (e.g., “I act charming or nice to get things I want”; α = .71–.73) subscales were used in analyses. There is substantial support for the validity of the self-report version of the APSD (Kimonis et al. 2006).

#### Proactive and Reactive Aggression (Time 1 and 2)

The self-rating scale of the *Proactive and Reactive Aggression Questionnaire* (Raine et al. 2006) is a 23-item questionnaire that measures proactive (12 items; e.g., “Had fights with others to show who was on top”; α = .89–.90) and reactive aggression (11 items; e.g., “Gotten angry when others threatened you”; α = .89). Items are rated on a 3-point Likert scale ranging from 0 (never) to 2 (often) for frequency of occurrence. The items refer either to physical or verbal aggression for both proactive and reactive aggression subscales.

#### Sensation Seeking (Time 2)

The *Sensation Seeking Scale Form*-*V* (SSS-V; Zuckerman 2003) is a 40-item forced choice questionnaire that was developed to measure individual differences in stimulation and arousal needs. The SSS-V yields four 10-item subscales: Thrill and Adventure Seeking, Experience Seeking, Disinhibition and Boredom Susceptibility. Scores are summed to form a total score (α = .80), which was used in the current study. The reliability, construct and cross-cultural validity for this instrument is well established (Zuckerman 1994).

#### Peer Relationships (Time 1)

The *Peer Pressure Questionnaire* (PPQ; Santor et al. 2000) is a 30-item self-report questionnaire that yields three subscales: The 7-item peer conformity subscale assesses the extent to which individuals are obedient and conform to authority in general (e.g., “I usually do what I am told”; α = .67); the 11-item peer pressure subscale assesses the subjective experience of feeling pressured, urged, or dared by peers to do certain things (e.g., “My friends could push me into doing just about anything”; α = .77); and the 12-item popularity striving subscale measures an individual’s intention to do certain things in order to be viewed as popular among their peers (e.g., “I have done things to make me more popular, even when it meant doing something I would not usually do”; α = .85). PPQ items, which are rated on a 5-point Likert scale ranging from 0 (strongly disagree) to 4 (strongly agree), were averaged to create the three subscales.

#### Media Violence Exposure (Time 1)

Based on prior work by Funk et al. (2004), participants were asked to report the average amount of time per week (ranging from 0 to more than 20 h) they were exposed to violent media content (TV, internet and movies at home or in movie theatres) and the time they spend playing violent video games (*α* = .89–.95). The measure was administered at Time 1. The measure demonstrated adequate reliability and validity in samples of adolescents (Fanti 2013; Fanti et al. 2013).

### Statistical Analyses

Group comparisons were computed using repeated measures ANOVAs including the five groups (low risk, callous–unemotional traits alone, increasing symptoms, conduct disorder symptoms alone and combined callous–unemotional and conduct disorder symptoms) as between-subject factor and the various individual, contextual, and personality variables measured longitudinally as dependent variables. For analysis with variables assessed cross-sectionally, one-way ANOVA was used. All analyses were run in SPSS (IBM, Armonk, NY: IBM). Alpha levels for post hoc tests were adjusted for multiple testing using Bonferroni correction.

## Results

### Change Across the Two Time Points

Repeated measures ANOVAs for callous–unemotional traits for the five groups revealed a significant group difference (*F*(4,2033) = 543.99, *p* < .001, *η*^2^ = .52) and a significant time effect (*F*(1,2033) = 63.58, *p* < .001, *η*^2^ = .03) as well as an interaction between time and group (*F*(4,2033) = 68.87, *p* < .001, *η*^2^ = .12). A similar analysis for conduct disorder symptoms for the five groups revealed a significant group difference (*F*(4,2033) = 359.10, *p* < .001, *η*^2^ = .41) and a significant time effect (*F*(1,2033) = 712.57, *p* < .001, *η*^2^ = .26) as well as an interaction between time and group (*F*(4,2033) = 359.10, *p* < .001, *η*^2^ = .41). In separate analyses per time point, post hoc tests for the group differences show for conduct disorder symptoms at Time 1 no difference between youth in the groups with low risk and with callous–unemotional traits alone, but all other groups differ from each other; for conduct disorder symptoms at Time 2, all groups are separated and youth in the “increasing” group scored highest compared to all other groups (see Fig. 1; Table 1). For callous–unemotional traits at Time 1, youth in the low risk group appeared as a separate group, while youth in groups with conduct disorder symptoms and with increasing symptoms did not differ significantly from each other and scored lower than youth in those groups with combined callous–unemotional and conduct disorder symptoms or callous–unemotional traits alone, who did not differ significantly from each other. However, for callous–unemotional traits at Time 2, youth in the low risk group scored lower than those in the group with conduct symptoms alone and both groups scored lower than youth in groups with increasing symptoms, combined callous–unemotional and conduct disorder symptoms or callous–unemotional traits alone (see Fig. 1).

**Table 1:** Descriptive variables for all five groups

	“Low” (*n* = 1537)		“CU-only” (*n* = 180)		“Increasing” (*n* = 111)		“CD + CU” (*n* = 114)		“CD-only” (*n* = 96)		*p*
Age	16.98 (0.91)^ab^		17.04 (0.91)^b^		16.73 (0.88)^a^		16.90 (1.03)^ab^		16.88 (0.77)^ab^		.04
Gender	585 m 943f^a^		112 m 66f^b^		93 m 17f^c^		97 m 15f^c^		66 m 29f^c^		<.001

	Time 1	Time 2	Time 1	Time 2	Time 1	Time 2	Time 1	Time 2	Time 1	Time 2	
YI-4 CD	1.87 (1.86)^a^	2.30 (2.16)^a^	2.61 (2.24)^a^	3.76 (2.45)^b^	3.98 (3.47)^b^	19.50 (8.66)^e^	16.56 (8.56)^d^	16.83 (8.11)^d^	8.90 (5.48)^c^	11.71 (7.14)^c^	<.001
ICU CU	19.99 (6.65)^a^	19.72 (6.53)^a^	34.15 (4.13)^b^	34.08 (4.04)^c^	25.25 (7.97)^c^	35.99 (5.76)^cd^	35.86 (6.49)^c^	36.18 (4.93)^d^	23.61 (5.32)^b^	22.93 (7.03)^b^	<.001

Means and standard deviations (*M*(*SD*)) as well as frequencies at measurement points (Time 1 and Time 2)

*YI-4* Checkmate plus Youth’s Inventory-4*, CD* conduct disorder, *ICU* inventory of callous–unemotional traits, *CU* callous–unemotional traits

*p* for group comparison, means with different subscripts (a, b, c, d) differ significantly from each other at the *p* < .05 level

### Internalizing Problems

**Fig. 2:**
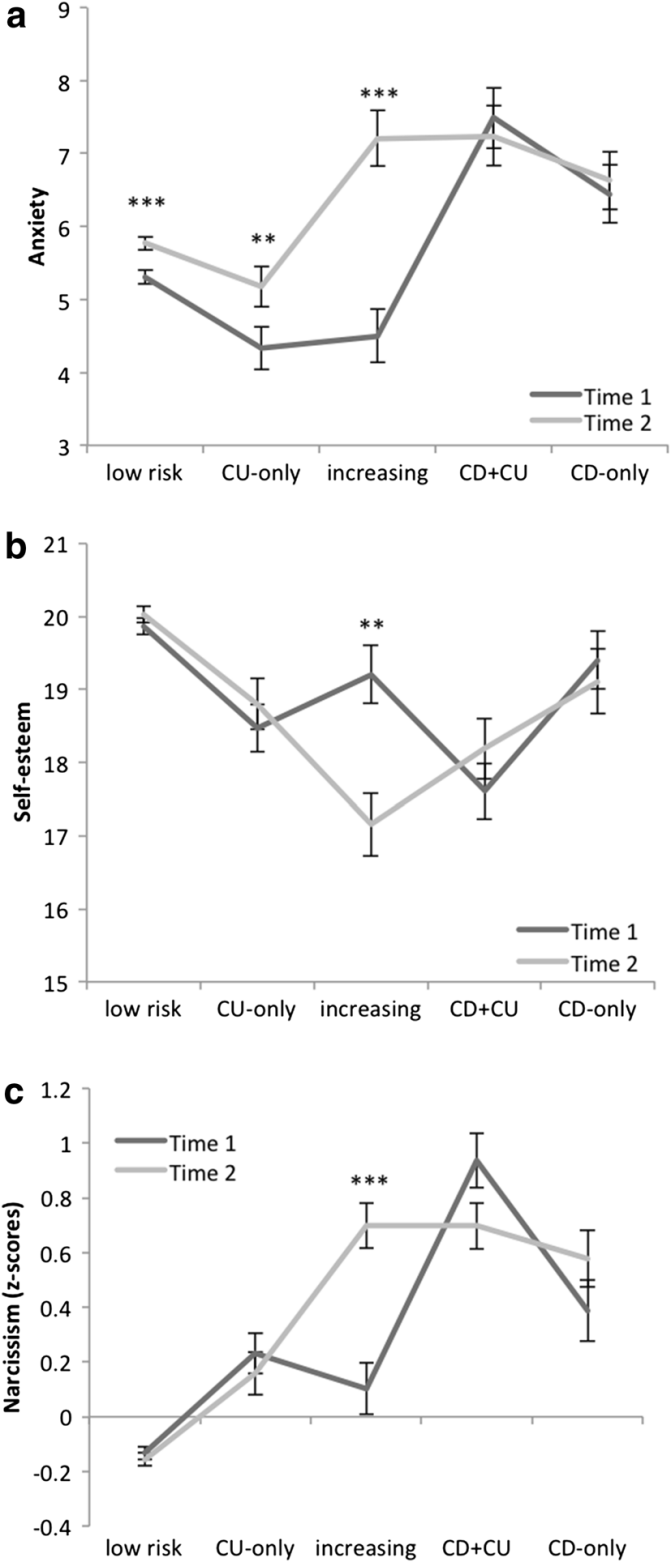
Anxiety (**a**), Self-esteem (**b**) and Narcissism (**c**, z-scored) scores at Time 1 and Time 2 for the 5 groups: “low risk”, “CU-only”, “increasing”, “CD + CU” and “CD-only”: means and standard errors of the mean, differences between Time 1 and Time 2 for single groups. **p < .05; ***p < .001

Comparing the identified groups on anxiety suggested the following: youth in the low risk group were significantly less anxious compared to youth in groups with combined callous–unemotional and conduct disorder symptoms or conduct disorder symptoms alone. Further, youth in the group with callous–unemotional traits alone were significantly less anxious compared to all other groups (see Table 2). A significant effect of time (*F*(1,2033) = 34.82, *p* < .001, *η*^2^ = .02) reflected an overall increase in anxiety scores. The group × time interaction (*F*(4,2032) = 12.49, *p* < .001, *η*^2^ = .02) suggested significant increase from Time 1 to Time 2 in anxiety for youth in groups with low risk, callous–unemotional traits alone and increasing symptoms, but not in the groups with combined callous–unemotional and conduct disorder symptoms or conduct disorder symptoms alone (see Fig. 2a). As shown in Fig. 2, youth in the group with increasing symptoms score similarly on anxiety as the groups with conduct disorder symptoms alone or combined callous–unemotional and conduct disorder symptoms and higher compared to the groups with callous–unemotional traits alone or low risk. The analysis comparing groups on depressive symptoms at Time 2 also resulted in significant differences across groups, (*F*(4,2032) = 44.92, *p* < .001, *η*^2^ = .08). Youth in the groups with callous–unemotional traits alone and low risk showed the lowest scores on depression compared to the other groups, with youth in the group with combined callous–unemotional and conduct disorder symptoms scoring higher on depression compared to youth with conduct disorder symptoms alone.

**Table 2:** Group comparison averaged across Time 1 and Time 2 for all measurements taken at both measurement points

	“Low” (n = 1537)	“CU-only” (n = 180)	“Increasing” (n = 111)	“CD + CU” (n = 114)	“CD-only” (n = 96)	*F*	*df*	*η* ^2^
	*M*(*SD*)	*M*(*SD*)	*M*(*SD*)	*M*(*SD*)	*M*(*SD*)			
Internalizing								
Anxiety	5.54 (0.09)^ab^	4.75 (0.29)^a^	5.85 (0.37)^b^	7.37 (0.41)^d^	6.54 (0.40)^cd^	12.72	4	.02
Depressive symptoms (T2)	8.98 (4.46)^a^	9.32 (4.49)^a^	12.91 (5.74)^bc^	13.31 (5.34)^c^	11.81 (5.33)^b^	44.92	4	.08
Aggression								
Proactive aggression	1.63 (0.05)^a^	2.97 (0.22)^b^	7.08 (0.41)^d^	9.55 (0.44)^e^	5.81 (0.40)^c^	563.88	4	.53
Reactive aggression	7.73 (0.09)^a^	8.29 (0.29)^a^	10.61 (0.41)^b^	13.14 (0.41)^d^	11.80 (0.39)^c^	126.10	4	.20
Personality								
Self-esteem	19.95 (0.11)^c^	18.64 (0.34)^ab^	18.18 (0.41)^ab^	17.90 (0.40)^a^	19.26 (0.42)^bc^	15.13	4	.03
Narcissism (z-scored)	−0.14 (0.02)^a^	0.20 (0.08)^b^	0.40 (0.09)^bc^	0.82 (0.09)^d^	0.48 (0.11)^c^	55.02	4	.10
Sensation seeking (T2)	16.28 (5.28)^a^	18.18 (5.34)^b^	19.21 (4.48)^bc^	21.38 (4.43)^d^	20.62 (4.75)^cd^	46.56	4	.08
Impulsivity (T1)	4.42 (2.45)^a^	5.20 (3.07)^ab^	5.34 (2.94)^b^	7.75 (3.69)^d^	6.26 (2.67)^c^	53.46	4	.10
Environmental								
Peer conformity (T1)	16.37 (3.89)^d^	13.48 (4.39)^bc^	14.68 (4.15)^c^	11.92 (4.70)^a^	12.87 (4.37)^ab^	62.82	4	.11
Popularity striving (T1)	11.66 (7.21)^a^	11.13 (7.21)^a^	11.92 (7.41)^a^	18.45 (8.85)^c^	15.72 (7.28)^b^	61.17	4	.11
Peer pressure (T1)	9.83 (5.54)^a^	11.12 (6.24)^a^	11.28 (6.12)^a^	17.54 (6.55)^c^	14.48 (5.35)^b^	61.17	4	.11
Media violence exposure (T1)	12.04 (7.35)^a^	13.63 (7.96)^a^	16.46 (10.97)^b^	23.29 (11.63)^c^	16.49 (8.35)^b^	62.50	4	.11

Means and standard deviations (*M*(*SD*)) across both measurement points, unless otherwise specified. T1 is used to indicate variables only measured at Time 1 and T2 at Time 2; *F* and *η* ^2^ values for group comparison, means with different subscripts differ significantly from each other at the *p* < .05 level

### Aggressive Behavior

**Fig. 3:**
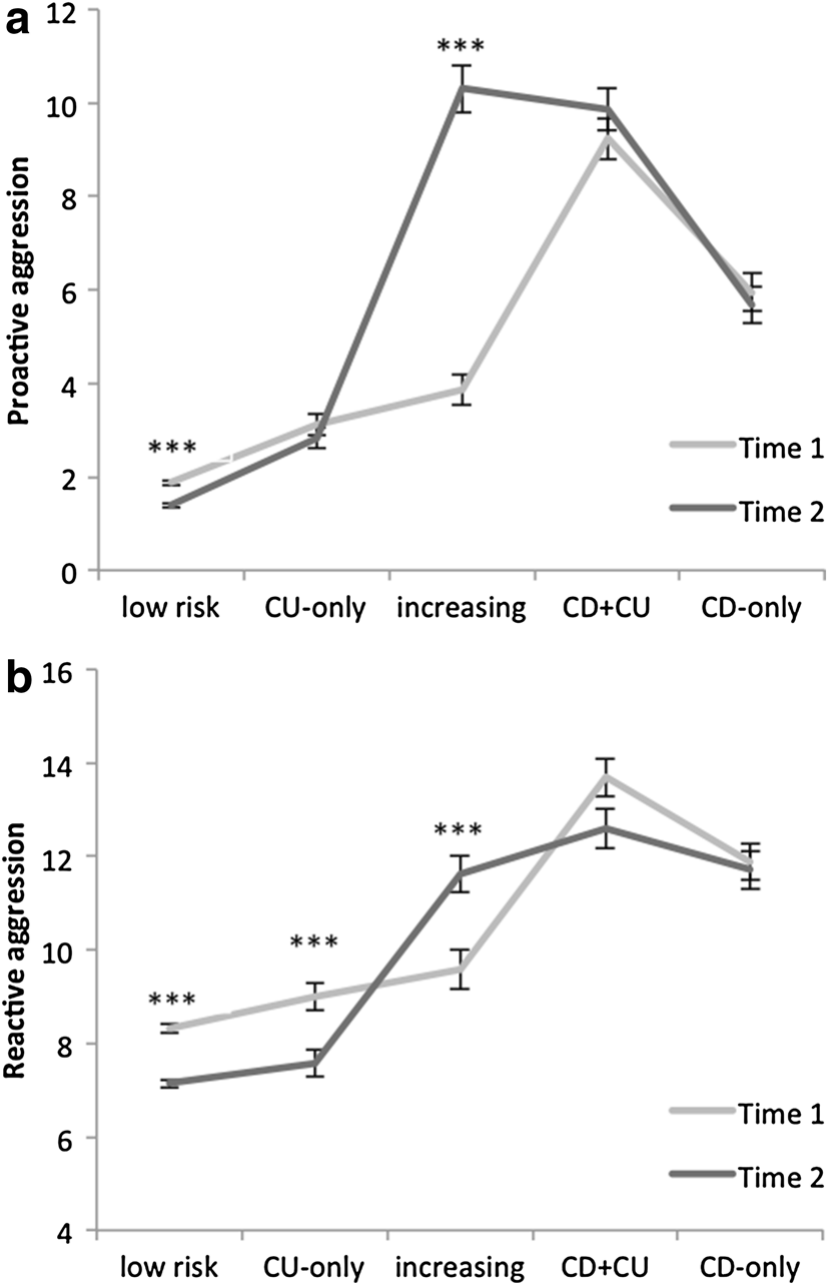
Proactive (**a**) and reactive (**b**) aggression scores at Time 1 and Time 2 for the 5 groups “low risk”, “CU-only”, “increasing”, “CD + CU” and “CD-only”: means and standard errors of the mean, differences between Time 1 and Time 2 for single groups. ***p < .001

For proactive aggression, we also found a main effect of group, with all groups differing significantly from each other. Youth in the low risk group had the lowest scores, while youth in the combined callous–unemotional and conduct disorder symptoms group the highest (see Table 2). A main effect of time revealed an overall increase of proactive aggression across groups (*F*(1,2033) = 108.09, *p* < .001, *η*^2^ = .05). The time × group interaction (*F*(4,2033) = 128.97, *p* < .001, *η*^2^ = .20) reflected a significant decrease in proactive aggression from Time 1 to Time 2 in youth in the low risk group and a significant increase of proactive aggression for youth in the group with increasing symptoms (see Fig. 3a).

For reactive aggression, a main effect of groups suggested that youth in the groups with low risk and with callous–unemotional traits alone showed the lowest scores and those in the combined callous–unemotional and conduct disorder symptoms group the highest scores (see Table 2). A main effect of time however, reflected an overall decrease in reactive aggression across groups (*F*(1,2033) = 7.44, *p* = .01, *η*^2^ = .004), whereas the interaction of group × time (*F*(4,2032) = 20.72, *p* < .001, *η*^2^ = .04) was based on a decrease in reactive aggression scores for youth in groups with low risk and with callous–unemotional traits alone as well as an increase among youth in the group with increasing symptoms (see Fig. 3b).

### Personality

Four personality traits were assessed: Self-esteem and narcissism were measured across time, while sensation seeking was measured at Time 2 and impulsivity at Time 1. For self-esteem, we found a main effect of group with youth in the low risk group showing significantly higher self-esteem compared to youth in groups with callous–unemotional traits alone, increasing or combined callous–unemotional and conduct disorder symptoms, while youth in the group with combined callous–unemotional and conduct disorder symptoms showing significantly lower self-esteem compared to all other groups (see Table 2). An interaction of group × time (*F*(4,2033) = 8.85, *p* < .001, *η*^2^ = .02) reflected a decrease of self-esteem from Time 1 to Time 2 among youth in the “increasing” group, while all other groups remained stable (see Fig. 2b).

Similarly, we found a main effect of group for narcissistic traits, with youth in the low risk group scoring lower compared to all other groups, while youth in the group with combined callous–unemotional and conduct disorder symptoms showed the highest scores on narcissism (see Table 2). A main effect of time (*F*(1,2033) = 7.79, *p* = .01, *η*^2^ = .004) reflected an overall increase of narcissism scores from Time 1 to Time 2. The interaction of group × time (*F*(4,2033) = 14.56, *p* < .001, *η*^2^ = .03) was driven by a significant increase of narcissism scores in the group with increasing symptoms (see Fig. 2c).

Furthermore, groups differed significantly on sensation-seeking, with lowest scores among youth in the low risk group compared to all other groups and highest scores among youth in the group with combined callous–unemotional and conduct disorder symptoms compared to all other groups. In terms of impulsivity, the lowest scores were identified for youth in the low risk group, followed by youth in the group with callous–unemotional traits alone. The combined group scored higher compared to the increasing group, and similarly as the group with stable high conduct disorder symptoms alone (see Table 2).

### Contextual Measures

All contextual measures included in the current study were assessed at Time 1 and findings are reported in Table 2. Youth in the identified groups varied on peer conformity, with highest scores identified for youth in the low risk group compared to all other groups and the lowest scores for youth in the group with combined stable high callous–unemotional and conduct disorder symptoms. Findings for popularity striving suggested that youth in groups with combined callous–unemotional and conduct disorder symptoms or conduct disorder symptoms alone scored higher compared to all other groups. Youth in the low risk group also had the lowest scores on peer pressure compared to all other groups except the group with increasing symptoms, while youth in groups with combined callous–unemotional and conduct disorder symptoms or conduct disorder symptoms alone reported the highest scores. Finally, groups also differed on media violence exposure, with highest scores reported among youth in the group with combined callous–unemotional and conduct disorders symptoms compared to all other groups and lowest scores among youth in the low risk group and the group with callous–unemotional traits alone.

## Discussion

Callous–unemotional traits and conduct disorder symptoms tend to co-occur across development (Fanti and Centifanti 2014; Klingzell et al. 2015). Although it is relevant for the development of targeted interventions, their stability during adolescence is still unclear. The current study provides evidence for the existence of groups showing stable high conduct disorder symptoms with or without callous–unemotional traits, providing evidence for heterogeneity in adolescent antisocial behavior. Additionally, a group of youth high on callous–unemotional traits but low on conduct disorder symptoms was identified, indicating that despite low levels of empathy these children do not engage in antisocial behaviors. Providing evidence for change over time, a group of adolescents demonstrating increases in both conduct disorder and callous–unemotional traits has been identified. Importantly, a decreasing group was not identified, suggesting that during the adolescent development period these characteristics and behaviors tend to be more stable.

Results from the current study were largely consistent with a priori hypotheses, highlighting specific correlates of the stability subtypes. First, the low antisocial behavior of youth in the group with stable high callous–unemotional traits alone might be explained by their low levels of peer pressure, popularity striving, sensation seeking and media violence exposure. This group also was at low risk in terms of anxiety, depression, proactive and reactive aggression, but not narcissism. Second, increases in both conduct disorder symptoms and callous–unemotional traits shown by the “increasing” group were associated with high levels of anxiety, reactive/proactive aggression, narcissism and lower levels of self-esteem. Third, children with stable conduct disorder symptoms, irrespective of callous–unemotional traits, scored high on measures of reactive aggression, anxiety and sensation seeking demonstrating that these measures do not differentiate heterogeneous antisocial subgroups. As a result, emotional dysregulation and high sensation seeking might make both groups vulnerable to engaging in antisocial behaviors. Adolescents with stable high callous–unemotional and conduct disorder problems were the ones scoring higher on proactive aggression, narcissism and lower on self-esteem and were more likely to be exposed to media violence, to experience peer pressure and to strive for peer conformity and popularity. These findings indicate that both individual and contextual maladjustment might explain the co-development of conduct disorder symptoms and callous–unemotional traits.

### The High Stable Callous–Unemotional Group as a Low-Risk Group

In contrast to youth with both stable high callous–unemotional traits and conduct disorder symptoms, those with only stable high callous–unemotional traits scored low on anxiety and reactive aggression, indicating that higher emotional and behavioral regulation might protect them from engaging in antisocial behaviors. This could be an indicator for a pathway to so-called successful psychopathy. As described recently by Lilienfeld et al., “the *successful psychopath*, sometimes termed the *adaptive* or *subclinical psychopath[is] an individual who displays many of the core features of psychopathic personality (psychopathy) while achieving success*” (Lilienfeld et al. 2015, p. 298). One of the suggested models of successful psychopathy states that it is related to a more intact autonomic activity compared to non-successful psychopaths (Ishikawa et al. 2001) and an even superior cognitive control functioning (Gao et al. 2011), which could be protective from engaging in antisocial behavior. Our findings point to such a protective aspect associated with the group of adolescents showing stability in callous–unemotional traits with low conduct disorder symptoms.

### The Non-stable High-Risk Group

The most striking results are related to the non-stable “increasing” group. Compared to stability subtypes in childhood, in our adolescent sample, we found no evidence for a group that shows a decrease of conduct disorder symptoms and callous–unemotional traits over the time of the assessment period (see Fanti and Centifanti 2014; Klingzell et al. 2015). Thus, it could be hypothesized that these traits become more stable during adolescence, and that the “increasing” subtype is still emerging in adolescence. Youth in this group show a pattern, which changes from low risk to a combined type, ending with high scores in both callous–unemotional traits and conduct disorder symptoms. Compared to youth with stable conduct disorder symptoms, those in the “increasing” group were characterized by high peer conformity, low popularity striving, and low peer pressure at Time 1. The increasing group also showed longitudinal increases in anxiety and narcissism, decreases in self-esteem, and reported high sensation seeking and depressive symptoms at Time 2. These findings suggest that increases in internalizing problems and emotional dysregulation, but not peer relationships, drive changes in conduct disorder symptoms and callous–unemotional traits. Further, increases in narcissism and decreases in self-esteem might result in a combination associated with defensive egotism that leads to the engagement in aggressive behavior (Baumeister and Heatherton 1996; Fanti and Henrich 2015). Indeed, youth in the increasing group demonstrated increases in both proactive and reactive forms of aggressive behavior. Thus, this is an important group of adolescents, since in a short period of time they come to resemble youth from the group with both stable high callous–unemotional traits and conduct disorder symptoms in terms of antisocial and aggressive behavior, possibly placing them at similar risk for adult criminal and antisocial behavior (see Frick et al. 2014 for a review).

### The Usual Suspects Group: High Callous–Unemotional and Conduct Disorder Symptoms

Youth with both stable high callous–unemotional traits and conduct disorder symptoms were the ones scoring high on proactive aggression, agreeing with theory and research suggesting that these individuals use planned and manipulative forms of aggressive behavior (Feilhauer et al. 2012; Roose et al. 2010). High levels of narcissism and low levels of self-esteem differentiated those with both high callous–unemotional traits and conduct disorder symptoms from those with only stable high conduct disorder symptoms. Fanti (2013) also found that those with both high callous–unemotional traits and conduct disorder symptoms tend to be more narcissistic with low levels of self-esteem and suggested that this combination might reflect maladaptive narcissism that has been associated with high levels of aggressive behavior (Fanti and Henrich 2015).

The high levels of narcissism within the group with both high callous–unemotional traits and conduct disorder symptoms might also explain why they report higher popularity striving as a mean of social acceptance. On the other hand, their low self-esteem might make them vulnerable to high peer pressure. Interestingly, youth in this group were more likely to be exposed to media violence, which is a known correlate of antisocial behavior and psychopathic traits (Fanti, 2013).

### The Stable High Conduct Disorder Symptoms Group Without Callous–Unemotional Traits

Our findings confirm previous results on the difference between adolescents with conduct disorder symptoms combined with high versus low callous–unemotional traits: Youth in our sample with conduct disorder symptoms but low callous–unemotional traits reported lower proactive aggression but similar reactive aggression compared to youth with combined symptoms as well as higher self-esteem (Fanti, 2013). However, extending previous reports, we found lower violent media exposure and less peer pressure reports in this group, pointing to less environmental stressors. These findings provide unique evidence that the youth with conduct disorder problems alone and those with both stable high callous–unemotional traits and conduct disorder problems can be differentiated on peer related measures, which are important for adjustment during adolescence. Although anxiety did not differentiate the two conduct disorder groups, the group high on conduct disorder alone was more likely to report high levels of depression than the combined group.

### Strengths and Limitations

The longitudinal nature of this study offers a new perspective on stability types based on callous–unemotional traits and conduct disorder symptoms in adolescents. The detection of one unstable group and the comparison with stable groups showing different combinations of callous–unemotional and conduct disorder symptoms for personality related and environmental differences has a strong impact on the understanding of the development of those symptoms. Another strength of this study is the inclusion of personality related variables and environmental variables, such as violent media exposure and peer relationships. Furthermore, the dataset is fairly large and provides sufficient power. However, interpretations from our data have to be derived carefully as the data are based on a rather short follow-up period, with one-year difference between the two time points. In addition, the data are only based on self-report questionnaires. It will be important to compare the stable and increasing groups on psychophysiological and neuropsychological measures.

## Conclusions

Our findings provide important developmental information on both stable and unstable subtypes of callous–unemotional traits and conduct disorder symptoms and their combination. We were able to describe the high functionality of youth showing stable callous–unemotional traits without conduct disorder symptoms as well as a strong drop in functionality (high aggression, low self-esteem) in youth with increasing callous–unemotional and conduct disorder symptoms. Thus, stability subtypes might help to understand developmental pathways of callous–unemotional traits and conduct disorder symptoms beyond cross-sectional group analyses and should be considered in future research. Finally, findings provide evidence for the Limited Prosocial Emotions specifier for conduct disorder in the Diagnostic and Statistical Manual 5 edition (DSM-5), suggesting possible variables associated with similarities and differences between the two conduct disorder subtypes. The study also provides evidence that an adolescent onset group with both high callous–unemotional traits and conduct disorder symptoms influenced by peer related variables should also be considered.

## References

[CR1] Andershed H, Kerr M, Stattin H, Levander S, Blaauw E, Sheridan L (2002). Psychopathic traits in nonreferred youths: A new assessment tool. Psychopaths: Current international perspectives.

[CR2] Baumeister RF, Heatherton TF (1996). Self-regulation failure: An overview. Psychological Inquiry.

[CR3] Blair RR (2001). Neurocognitive models of aggression, the antisocial personality disorders, and psychopathy. Journal of Neurology, Neurosurgery and Psychiatry.

[CR4] Brummelman E, Thomaes S, Sedikides C (2016). Separating narcissism from self-esteem. Current Directions in Psychological Science.

[CR5] Dodge KA, Bates JE, Pettit GS (1990). Mechanisms in the cycle of violence. Science.

[CR6] Essau CA, Sasagawa S, Frick PJ (2006). Callous–unemotional traits in a community sample of adolescents. Assessment.

[CR7] Fanti KA (2013). Individual, social, and behavioral factors associated with co-occurring conduct problems and callous–unemotional traits. Journal of Abnormal Child Psychology.

[CR8] Fanti KA, Centifanti LM (2014). Childhood callous–unemotional traits moderate the relation between parenting distress and conduct problems over time. Child Psychiatry and Human Development.

[CR9] Fanti KA, Colins OF, Andershed H, Sikki M (2016). Stability and change in callous–unemotional traits: Longitudinal associations with potential individual and contextual risk and protective factors. American Journal of Orthopsychiatry.

[CR10] Fanti KA, Demetriou CA, Kimonis ER (2013). Variants of callous–unemotional conduct problems in a community sample of adolescents. Journal of Youth and Adolescence.

[CR11] Fanti K, Frick P, Georgiou S (2009). Linking callous–unemotional traits to instrumental and non-instrumental forms of aggression. Journal of Psychopathology and Behavioral Assessment.

[CR12] Fanti KA, Henrich CC (2015). Effects of self-esteem and narcissism on bullying and victimization during early adolescence. Journal of Early Adolescence.

[CR13] Fanti KA, Panayiotou G, Lombardo MV, Kyranides MN (2016). Unemotional on all counts: Evidence of reduced affective responses in individuals with high callous–unemotional traits across emotion systems and valences. Social Neuroscience.

[CR14] Feilhauer J, Cima M (2013). Youth psychopathy: Differential correlates of callous–unemotional traits, narcissism, and impulsivity. Forensic Science International.

[CR15] Feilhauer J, Cima M, Korebrits A, Kunert H-J (2012). Differential associations between psychopathy dimensions, types of aggression, and response inhibition. Aggressive Behavior.

[CR16] Fite PJ, Stoppelbein L, Greening L (2009). Proactive and reactive aggression in a child psychiatric inpatient population: Relations to psychopathic characteristics. Criminal Justice and Behavior.

[CR17] Fontaine NMG, McCrory EJP, Boivin M, Moffitt TE, Viding E (2011). Predictors and outcomes of joint trajectories of callous–unemotional traits and conduct problems in childhood. Journal of Abnormal Psychology.

[CR18] Frick PJ (2004). Inventory of callous–unemotional traits. Unpublished rating scale.

[CR19] Frick PJ, Cornell AH, Barry CT, Bodin SD, Dane HE (2003). Callous–unemotional traits and conduct problems in the prediction of conduct problem severity, aggression, and self-report of delinquency. Journal of Abnormal Child Psychology.

[CR20] Frick PJ, Ellis M (1999). Callous–unemotional traits and subtypes of conduct disorder. Clinical Child and Family Psychology Review.

[CR21] Frick PJ, Hare RD (2001). The antisocial process screening device.

[CR22] Frick PJ, Ray JV, Thornton LC, Kahn RE (2014). Annual research review: A developmental psychopathology approach to understanding callous–unemotional traits in children and adolescents with serious conduct problems. Journal of Child Psychology and Psychiatry.

[CR23] Funk JB, Baldacci HB, Pasold T, Baumgardner J (2004). Violence exposure in real-life, video games, television, movies, and the internet: Is there desensitization?. Journal of Adolescence.

[CR24] Gadow KD, Sprafkin J (1999). Youth’s Inventory-4.

[CR25] Gadow KD, Sprafkin J, Carlson GA, Schneider J, Nolan EE, Mattison RE (2002). A DSM-IV-referenced, adolescent self-report rating scale. Journal of the American Academy of Child and Adolescent Psychiatry.

[CR26] Gadow KD, Sprafkin J, Weiss M (2004). Adult self-report inventory-4 manual.

[CR27] Gao Y, Raine A, Schug RA (2011). P3 event-related potentials and childhood maltreatment in successful and unsuccessful psychopaths. Brain and Cognition.

[CR28] Helfritz LE, Stanford MS (2006). Personality and psychopathology in an impulsive aggressive college sample. Aggressive Behavior.

[CR29] Ishikawa SS, Raine A, Lencz T, Bihrle S, Lacasse L (2001). Autonomic stress reactivity and executive functions in successful and unsuccessful criminal psychopaths from the community. Journal of Abnormal Psychology.

[CR30] Kahn RE, Byrd AL, Pardini D (2013). Callous–unemotional traits robustly predict future criminal offending in young men. Law and Human Behavior.

[CR31] Kimonis ER, Frick PJ, Fazekas H, Loney BR (2006). Psychopathy, aggression, and the processing of emotional stimuli in non-referred girls and boys. Behavioral Sciences and the Law.

[CR32] Kimonis ER, Frick PJ, Skeem J, Marsee MA, Cruise K, Munoz LC (2008). Assessing callous–unemotional traits in adolescent offenders: Validation of the inventory of callous–unemotional traits. Journal of the International Association of Psychiatry and Law.

[CR33] Klingzell I, Fanti K, Colins O, Frogner L, Andershed A-K, Andershed H (2015). Early childhood trajectories of conduct problems and callous–unemotional traits: The role of fearlessness and psychopathic personality dimensions. Child Psychiatry and Human Development.

[CR34] Kyranides MN, Fanti KA, Panayiotou G (2016). The disruptive adolescent as a grown-up: Predicting adult startle responses to violent and erotic films from adolescent conduct problems and callous–unemotional traits. Journal of Psychopathology and Behavioral Assessment.

[CR35] Lilienfeld SO, Watts AL, Smith SF (2015). Successful psychopathy: A scientific status report. Current Directions in Psychological Science.

[CR36] Longman T, Hawes D, Kohlhoff J (2015). Callous–unemotional traits as markers for conduct problem severity in early childhood: A meta-analysis. Child Psychiatry and Human Development.

[CR37] Mathias CW, Stanford MS (2003). Impulsiveness and arousal: Heart rate under conditions of rest and challenge in healthy males. Personality and Individual Differences.

[CR38] Moffitt TE (1993). Adolescence-limited and life-course-persistent antisocial behavior: A developmental taxonomy. Psychological Review.

[CR39] Patrick CJ, Zempolich KA (1998). Emotion and aggression in the psychopathic personality. Aggression and Violent Behavior.

[CR40] Raine A (2002). Biosocial studies of antisocial and violent behavior in children and adults: A review. Journal of Abnormal Child Psychology.

[CR41] Raine A, Dodge K, Loeber R, Gatzke-Kopp L, Lynam D, Reynolds C (2006). The reactive–proactive aggression questionnaire: Differential correlates of reactive and proactive aggression in adolescent boys. Aggressive Behavior.

[CR42] Roose A, Bijttebier P, Claes L, Lilienfeld SO (2010). Psychopathic traits in adolescence: Associations with the revised reinforcement sensitivity theory systems. Personality and Individual Differences.

[CR43] Rosenberg M (1965). Society and the adolescent self-image.

[CR44] Santor D, Messervey D, Kusumakar V (2000). Measuring peer pressure, popularity, and conformity in adolescent boys and girls: Predicting school performance, sexual attitudes, and substance abuse. Journal of Youth and Adolescence.

[CR45] Scarpa A, Haden SC, Tanaka A (2010). Being hot-tempered: Autonomic, emotional, and behavioral distinctions between childhood reactive and proactive aggression. Biological Psychology.

[CR46] Scarpa A, Tanaka A, Haden SC (2008). Biosocial bases of reactive and proactive aggression: The roles of community violence exposure and heart rate. Journal of Community Psychology.

[CR47] Stanford MS, Houston RJ, Baldridge RM (2008). Comparison of impulsive and premeditated perpetrators of intimate partner violence. Behavioral Sciences & the Law.

[CR48] Zuckerman M (1994). Behavioral expressions and biosocial bases of sensation seeking.

[CR49] Zuckerman M (2003). Are there racial and ethnic differences in psychopathic personality? A critique of Lynn’s (2002) racial and ethnic differences in psychopathic personality. Personality and Individual Differences.

